# Some Curious Things That Happen to Our Domestic Animals

**Published:** 1882-04

**Authors:** 


Some Curious Things that Happen to our Domestic Animals.—We collect, through Dr. A. Lydtin, the following interesting notes from the contributions of various German veterinary surgeons.

Many of the cases are instructive, as showing our readers the possible mishaps which they may be summoned to remedy:

*Chronic Vomiting in an Ox.*—A fifteen-months-old ox vomited regularly its food. Being put on a bran-mash, it stopped; but began again with a return to hay. *Post mortem* showed a diverticulum in the œsophagus the size of a child's head, filled with dry hay and straw.

*A Similar Diverticulum*, with almost complete stenosis of the cardia, was found in an old cow which had also suffered from chronic vomiting.

*Stenosis of the Œsophagus* in a horse was brought about by an inflammatory hypertrophy(?) of the muscular wall.

*A Lung-Stomach Fistula.*—A cow suffered from chronic indigestion and intermittent tympanites. *Post mortem* showed a fistula between the first stomach, the diaphragm and the lung, produced by a rusty nail.

*Hair-pin in the Soft-Palate.*—A horse neither drank nor eat for six days. He was otherwise healthy. A hair-pin was at last found in his soft-palate. Upon removal, the animal took nourishment as usual.

*A Fatty Liver.*—In a fine old English stallion, which had only been fed on hay and oats, a very large and fatty liver was found, weighing over fifty pounds. There were hemorrhagic spots in it.

*Intersusception in a Horse.*—A horse had for several weeks suffered from constant indigestion and intermittent colic. *Post mortem* showed that the cœcum had been forced down into the colon.

*Tetanus from Unskillful Castration* was observed several times in young steers by Dr. Heizmann, of Messkirch.

*The Result of Clipping.*—A fine horse was clipped in the Fall of the year. Immediately after, a well-marked attack of pleuro-stholouos was observed, which lasted for four weeks.

*A Fibro-sarcoma* within the skull pressed upon the trigeminus, causing paralysis and complete atrophy of the muscles of mastication upon that side. The horse had to be killed.

